# Cost-effectiveness of resistance-guided therapy for *Mycoplasma genitalium* in Australia

**DOI:** 10.1038/s41598-024-63056-1

**Published:** 2024-06-04

**Authors:** Rabiah Al Adawiyah, Catriona S. Bradshaw, Lenka A. Vodstrcil, Christopher K. Fairley, Lei Zhang, Jason J. Ong

**Affiliations:** 1 School of Translational Medicine, Faculty of Medicine, Nursing and Health Sciences, Monash University, Melbourne, VIC Australia; 2 Melbourne Sexual Health Centre, Alfred Health, Melbourne, Australia; 3 Centre for Epidemiology and Biostatistics, Melbourne School of Population and Global Health, The University of Melbourne, Melbourne, Australia; 4 Clinical Medical Research Center, Children’s Hospital of Nanjing Medical University, Nanjing Medical University, 210008 Nanjing, Jiangsu China; 5 Faculty of Infectious Diseases, London School of Hygiene and Tropical Medicine, London, UK

**Keywords:** Health care economics, Public health

## Abstract

The recommended first-line treatment for *Mycoplasma genitalium* infections is azithromycin. However, the prevalence of macrolide resistance for *M. genitalium* has increased to more than 50% worldwide. In 2013, Australia introduced a resistance-guided therapy (RGT) strategy to manage *M. genitalium* infections. This study assesses the cost-effectiveness of the RGT approach compared to no RGT (i.e., without macrolide resistance profile test) in women, men who have sex with men (MSM), and men who have sex with women (MSW) in Australia. We constructed dynamic transmission models of *M. genitalium* infections in women, MSM, and MSW in Australia, each with a population of 100,000. These models compared the costs and quality-adjusted life-years (QALYs) gained between RGT and no RGT scenarios from a healthcare perspective over ten years. All costs are reported in 2022 Australian dollars (Australian $). In our model, RGT is cost saving in women and MSM, with the incremental net monetary benefit of $1.3 million and $17.9 million, respectively. In MSW, the RGT approach is not cost-effective, with an incremental cost-effectiveness ratio of -$106.96 per QALY gained. RGT is cost saving compared to no RGT for *M. genitalium* infections in women and MSM, supporting its adoption as the national management strategy for these two population groups.

## Introduction

The pooled prevalence of Mycoplasma genitalium in randomly selected samples from the general population was 1.3% (95% CI 1.0% to 1.8%, I^2^ 41.5%) for 2007 to 2015 in higher Human Development Index (HDI) countries^1^. In many countries, *M. genitalium* was the second most prevalent bacterial sexually transmitted infection (STI) after *Chlamydia trachomatis*^2,3^. According to the third National Survey of Sexual Attitudes and Lifestyle (Natsal-3), the prevalence of *M. genitalium* was 1.2% and 1.3% in British men and women, respectively^4^. In the US, a multicentre surveillance study from sexual health clinics showed the overall prevalence of *M. genitalium* infection was 16.6% (95% CI 14.9–18.5%; site-specific range: 9.9–23.5%)^5^. In Australia*,* the prevalence of *M. genitalium* infections ranges from 1.3% to 3.9% in the community^1^, and 1.3% in 2017 in women^6^. A cross-sectional study in Australia on asymptomatic men who have sex with men (MSM) found that the prevalence rate of *M. genitalium* was 9.5% (95% CI 7.7% 0 11.5%)^7^. While *M. genitalium* infection is frequently asymptomatic, it can cause urethritis in men, and cervicitis, endometritis, and pelvic inflammatory disease (PID) in women, although these data are limited^8–10^. Research from Australia also suggests that *M. genitalium* could contribute to symptomatic proctitis, although data are conflicting between published studies^11,12^.

Current national and international guidelines for managing *M. genitalium* do not advise screening for *M. genitalium* in asymptomatic individuals^13,14^, largely because treatment is becoming increasingly challenging due to rising antimicrobial resistance (AMR)^15^. The recommended first-line treatment for *M. genitalium* infections globally has been 1 g of azithromycin, resulting in more than 10% of susceptible infections developing selected macrolide resistance^16^. Widespread use of 1 g azithromycin for STI syndromes, chlamydia, *M. genitalium* and *N. gonorrhoeae* is likely to have contributed to the rise in macrolide resistance in *M. genitalium*, particularly in MSM who have higher rates of STIs and high levels of antibiotic consumption^16–18^. In Australia, the proportion of diagnosed infections with macrolide resistance mutations (MRMs) increased from 18.8% in 2010 to 66.0% in 2016–2017^19^. *M. genitalium* infections are now commonly macrolide-resistant, and the proportion of infections with MRMs ranges from 32.7 to 44.0% in women, 53.0% in men who have sex with women (MSW), and 84.2–87.0% in MSM^16–18^.

Resistance-guided therapy (RGT) involves selecting antibiotics based on the macrolide resistance profile of each infection^20^. This strategy was developed to reduce the empiric use of azithromycin, enabling a more precise selection of first-line antibiotics, which improves antibiotic stewardship and the cure rate of first-line treatment. If doxycycline is used to treat STI syndromes instead of azithromycin, then when the macrolide resistance profile is known, macrolide-susceptible *M. genitalium* infections can be effectively treated with higher doses of azithromycin. Macrolide-resistant infections can be treated with moxifloxacin as a first-line treatment^16^. In a 2016 study, RGT resulted in cure rates of over 96% for *macrolide-susceptible* infections and 92% for *macrolide-resistant* infections and a reduction in selected macrolide resistance to less than 4% with the higher azithromycin dose^16^. As the incidence of AMR in *M. genitalium* continues to rise, using an RGT approach will promote antibiotic stewardship and could be less expensive than presumptive approaches^21^. Despite this potential, no economic evaluation of RGT for *M. genitalium* infections exists. To address this gap, this study aims to assess the cost-effectiveness of RGT compared to no RGT, where there is no macrolide resistance profile test performed for women, MSM and MSW living in Australia.

## Methods

### Model

**Figure 1:**
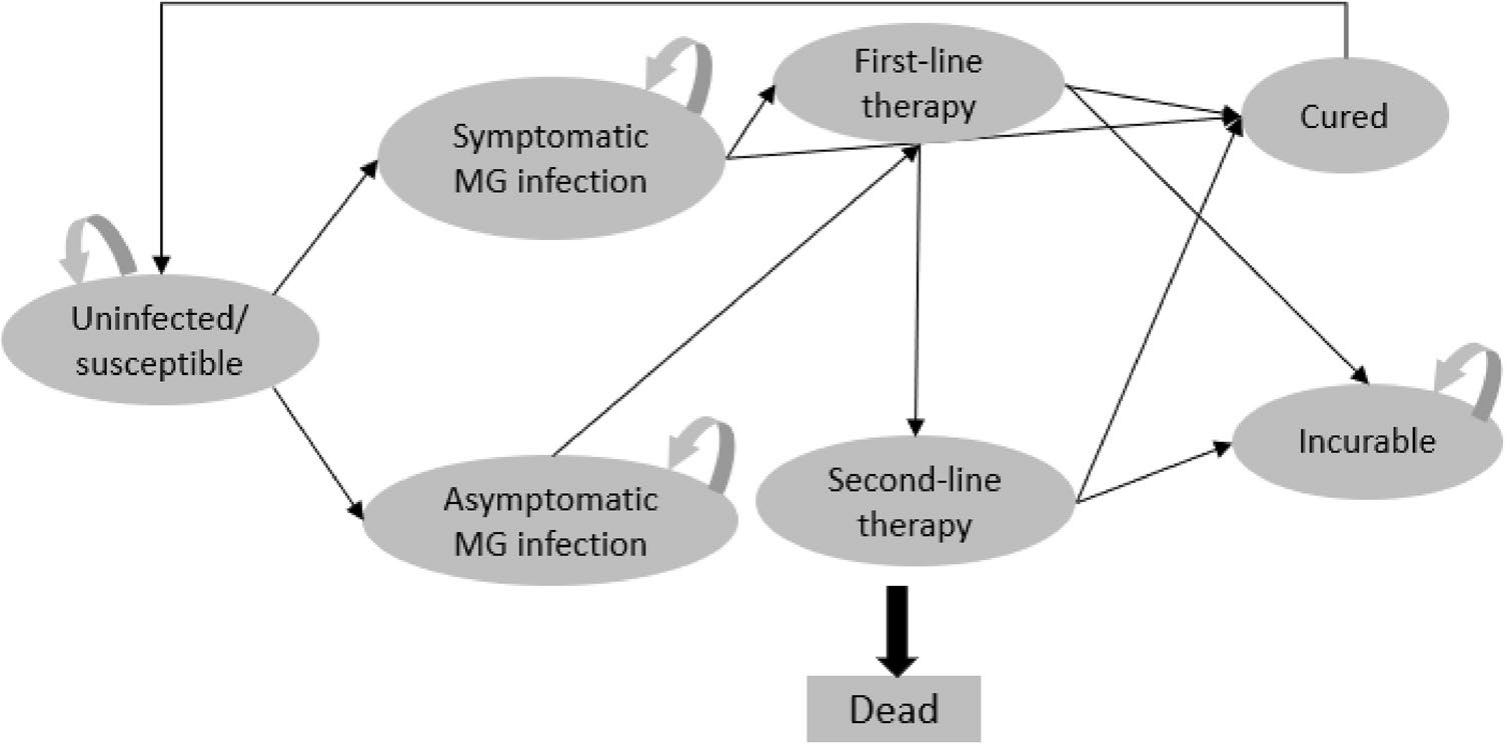
The dynamic transmission model of *M. genitalium* infections. *MG: *Mycoplasma genitalium;* In all health states, individuals had a probability of natural death and entered into a terminal state, “Dead”, as shown with a larger black arrow in the figure.

Dynamic transmission models of *M. genitalium* infection were constructed using TreeAge Pro Healthcare 2022 (Supplementary Fig. 1). There are three models that evaluated cohorts of 100,000 women, 100,000 MSM, and 100,000 MSW in Australia over ten years. The prevalence of *M. genitalium* varies in these three different population groups and is likely to be higher in specific populations whose behaviour puts them at a high risk of STI, such as MSM^6^. These three groups were selected to reflect the different risks of getting *M. genitalium* infections, the different rates of MRMs and consequent morbidity. All three models used a ten-year time horizon to capture the long-term complications of *M. genitalium* infections in women^10^. These three models used the same structure with different input parameters adjusted to each population group (Table 1). These three models were stratified by symptom status, distinguishing between symptomatic and asymptomatic *M. genitalium* infections (Fig. 1). The force of infection, which represents the rate at which susceptible individuals in the model became infected with *M. genitalium* at a given point in time, was determined as a function of the number of infectious individuals in a year (based on the *M. genitalium* infections incidence rate) and the population size of our cohort ^22^.

**Table 1:** Input parameters and ranges used in sensitivity analyses.

Parameters (unit)	Value	Range for univariate sensitivity analysis	Range for probability sensitivity analysis	References
The annual incidence rate of *M. genitalium* (per 100 person/year)	Women: 1.33	Women: 0.8–2.3	Not applicable	^22^
	MSM: 6.63	MSM: 6.63–29.5		^33^
	MSW: 1.3	MSW: 0.8–2.3		
Proportion cured using the first-line treatment (no RGT arm)	Women: 0. 54	Women: 95%CI (0.432–0.653)	Beta distribution Women: α 42.23, β 35.98	^19^
	MSM: 0.31	MSM: 95%CI (0.15–0.48)	MSM: α 9.27, β 19.79	
	MSW: 0.46	MSW: 95%CI (0.33–0.60)	MSW: α 22.86, β 26.83	
Proportion cured using the second-line treatment (no RGT arm)	0.85 (for all)	95% CI (0.8–0.89) (for all)	Beta distribution	^31^
			All: α 223.07, β 39.36	
Proportion cured using the first-line treatment (RGT arm)	0.93 (for all)	95% CI (0.87–0.98) (for all)	Beta distribution	^16^
			All: α 64.71, β 3.98	
Proportion cured using the second line treatment (RGT arm)	0.71 (for all)	95% CI (0.54–0.85) (for all)	Beta distribution	^34^
			All: α 22.71, β 9.28	
Spontaneous clearance rate (per week)	Women: 0.015	Not applicable	Not applicable	^15^
	MSM: 0.02			
	MSW: 0.02			

Costs input	Value (in 2022 prices, Australian $)	Range for univariate sensitivity analysis	Range for probability sensitivity analysis	References
Cost of GP visit	AU$ 38.75 (for all)	± 30% (AU$ 27.125–50.375)	Gamma distribution	MBS online
			All: α 43.14, γ 1.11	
Cost to diagnose *M. genitalium* infections	AU$ 28.65 (for all)	± 30% (AU$ 20.1–37.25)	Gamma distribution	MBS online
			All: α 42.88, γ 1.50	
Cost of resistance test	AU$ 11 (for all)	± 30% (AU$ 7.7–14.3)	Gamma distribution	MBS online
			All: α 1.8, γ 0.38	
Cost of 1st line treatment in no RGT	AU$ 17.38 (for all)	± 30% (AU$ 12.2–22.6)	Gamma distribution	PBS online
			All: α 42.69, γ 2.46	
Cost of 2nd line treatment in no RGT	AU$ 86.10 (for all)	± 30% (AU$ 60.27–111.93)	Gamma distribution	PBS online^35^
			All: α 42.67, γ 0.49	
Cost of 1st line in RGT	Macrolide resistance: AU$ 107.15 (for all)	± 30% (AU$ 75.01–139.64)	Gamma distribution	PBS online^35^
			All: α 42.70, γ 0.58	
	Macrolide susceptible: AU$ 73.19 (for all)	± 30% (AU$ 51.23- 95.15)	Gamma distribution	
			All: α 42.27, γ 0.39	
Cost of 2nd line in RGT (A proportion of those received Moxifloxacin and Minocycline)	AU$ 45.80 (for all)	± 30% (AU$ 32.06—59.54)	Gamma distribution	PBS online Calculation
			All: α 42.81, γ 0.93	

Utility input	Value	Range for univariate sensitivity analysis	Range for probability sensitivity analysis	References
Utility during *M. genitalium* infections	Women: 0.96	Women: ± 30% (0.67–1.0)	Beta distribution Women: α 4.80, β 0.20	^2^
	MSM: 0.96	MSM: ± 30% (0.67–1.0)	MSM: α 4.80, β 0.20	
	MSW: 0.96	MSW: ± 30% (0.67–1.0)	MSW: α 4.80, β 0.20	

CI: Confidence Interval; MBS: Medicare Benefits Schedule; MSM: Men who have sex with men; MSW: Men who have sex with men; PBS: Pharmaceutical Benefits Scheme; PSA: Probability Sensitivity Analysis; RGT: Resistance-Guided Therapy.

Treatment pathways were modelled, with only those in the symptomatic *M. genitalium* infection state transitioning to first-line therapy. Cohorts that failed the first-line therapy were transitioned to the second-line therapy. Cohorts in the model who did not complete treatment or were lost to follow-up during any treatment phase were transitioned to the incurable state. The incurable state was not regarded as terminal; cohorts within it could be cured, returning to an 'uninfected or susceptible state’ based on the weekly spontaneous cure rate^15^. Our model also allows *M. genitalium* infection, symptomatic or asymptomatic, to spontaneously clear without testing and treatment^15,23^. Figure 1 illustrates the structure of the model for symptomatic and asymptomatic *M. genitalium* infections in women, MSM and MSW. Cohorts in all health states in this model were subject to natural background mortality. The main parameters used in this model are shown in Table 1, and a complete list is available in Supplementary Table 1.

The model also measured *M. genitalium*-related complications in women, including PID and chronic pelvic pain^10^. A separate decision tree model was created to calculate the cumulative expected values of the probability, costs, and disutility associated with *M. genitalium*-related complications in women (Supplementary Fig. 2). This value was then assigned in the dynamic model with each new case of *M. genitalium*-related complications.

### Definition of scenarios

The study presents a comparison of RGT and no RGT for the treatment of *M. genitalium* infections (Supplementary Table 2). We acknowledge that the reference regimen for no RGT may differ globally from 1 g of azithromycin in many low- and middle-income countries, 1.5 g in some northern European countries, to even using moxifloxacin in some sub-populations where macrolide resistance is known to be high. For our model, we defined the reference scenario as no RGT and first-line therapy as azithromycin 1 g on day 1. If a patient fails this regimen, second-line therapy comprises 400 mg of moxifloxacin daily for seven days^14^.

In the RGT arm, the first-line therapy is based on macrolide susceptibility. For patients with macrolide-susceptible infections, the first-line therapy consists of doxycycline 100 mg twice a day for seven days, followed by azithromycin (1 g on day one and 500 mg for the next three days, totalling 2.5 g) for four days^14^. In contrast, the first-line therapy for patients with macrolide-resistant infections was doxycycline 100 mg twice daily, followed by moxifloxacin 400 mg daily for seven days. The second-line therapy consists of 400 mg of moxifloxacin daily for seven days for those who are macrolide-susceptible, and minocycline 100 mg twice a day for 14 days for those with macrolide-resistant.

### Cost-effectiveness analysis

The cost-effectiveness analysis was conducted from a healthcare provider’s perspective, following the Consolidated Health Economic Evaluation Reporting Standards (Supplementary Table 3)^24^, using a 3% annual discount rate for both costs (2022 Australian $) and units of health (quality-adjusted life-years [QALYs]). We compared the cost-effectiveness of RGT to the absence of RGT (no RGT) in treating *M. genitalium* infection. Transition probabilities, costs, and utilities in the model were determined using information from published literature and expert opinion (Table 1).

The model ran in a weekly cycle (total of 520 cycles), and therefore, the annual incidence and clearance rates for *M. genitalium* infection were transformed into weekly rates. Cure rates for the first-, and second line for RGT and no RGT were used as transition probabilities between treatment phases. When there was no published data on *M. genitalium* infection, parameters from chlamydia studies were utilised as a substitute. For instance, the proportion of symptomatic and asymptomatic *M. genitalium* infections in women was obtained from a published chlamydia study^25^. *Chlamydia trachomatis* and *M. genitalium* are highly prevalent bacterial STIs with a significant co-infection rate in some populations. Although caused by entirely different microorganisms, they share similarities in pathogenesis, clinical manifestations and treatment^26^.

The costs evaluated in this study were from a healthcare perspective and were calculated based on the unit costs from the Medicare Benefits Schedule (MBS) and Pharmaceutical Benefits Schedule (PBS). Direct medical costs were included in the model, such as costs associated with *M. genitalium* diagnosis and resistance testing, treatment, and medical visits. Costs for diagnosis include one consultation and one test for *M. genitalium* infections, while costs for treatment include one consultation and one for receiving treatment. Costs for additional medical visits or treatment for individuals in the incurable state were not added as no data were available for this cohort. Cumulative costs for *M. genitalium*-related complications were obtained from published evidence and converted to Australian dollars in 2022. All costs are reported in 2022 Australian dollars. Quality adjusted life years (QALYs) gained were estimated by applying utility (health-related) weights of the various health states. Data on the utility weights associated with *M. genitalium* infections were limited, and for women, a utility weight of 0.96 was assumed based on a previous cost-effectiveness study on chlamydia^27^. This weight was derived from the possibility of stress and anxiety resulting from a positive diagnosis. For MSM and MSW, a utility weight of 0.96 was used based on a published cost-effectiveness study on *M. genitalium* infections in MSM^15^.

### Sensitivity analysis

**Figure 2:**
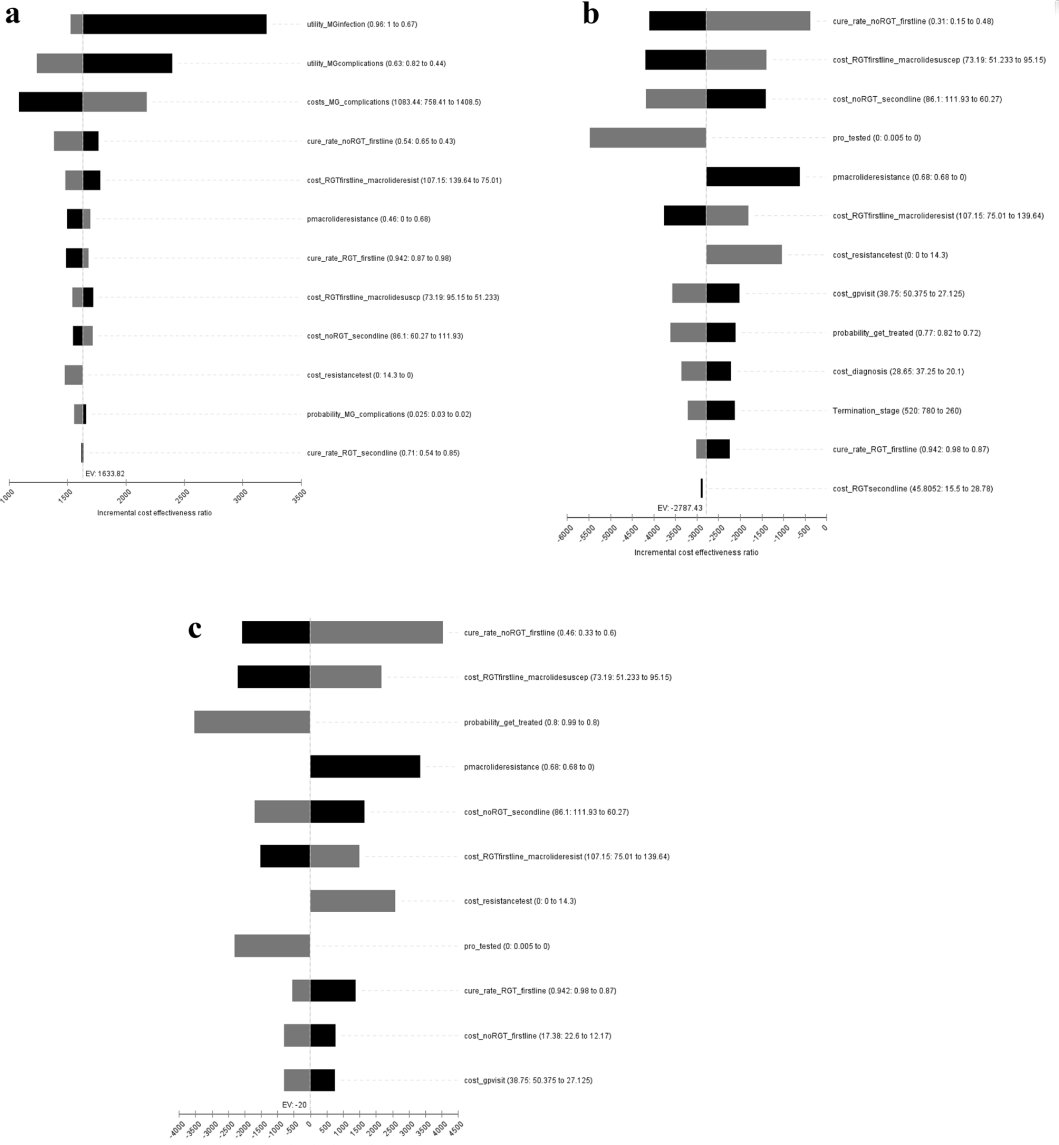
(**a**) Univariate sensitivity analysis of the Incremental Cost-Effectiveness Ratio of no RGT compared to RGT for *M. genitalium* infections in the cohort of 100,000 women. Black bars correspond to the effect of the low value in the sensitivity analysis, and grey bars correspond to the high value in the sensitivity analysis. This figure includes only the 12 variables with the highest uncertainty values. (**b**) Univariate sensitivity analysis of the Incremental Cost-Effectiveness Ratio of no RGT compared to RGT for *M. genitalium* infections in the cohort of 100,000 MSM. Black bars correspond to the effect of the low value in the sensitivity analysis, and grey bars correspond to the high value in the sensitivity analysis. This figure includes only the 12 variables with the highest uncertainty values. MSM, Men who have sex with men. (**c**)***:*** Univariate sensitivity analysis of the Incremental Cost-Effectiveness Ratio of no RGT compared to RGT for *M. genitalium* infections in the cohort of 100,000 MSW. Black bars correspond to the effect of the low value in the sensitivity analysis, and grey bars correspond to the high value in the sensitivity analysis. This figure includes only the 12 variables with the highest uncertainty values. MSW, Men who have sex with women.

Univariate sensitivity analyses were performed to assess the impact of uncertainties associated with the model parameters. Uncertainties in this study may arise from the data through natural variation in the population, from the evaluative process through generalising from the context of one study to other contexts and patient populations or from choice of analytical method. The following variables (rate of *M. genitalium* infections, probability of getting tested and treated, discount rate, time horizon, proportion of asymptomatic and symptomatic *M. genitalium* infections, probability of complications, and all costs included in the model) were varied over plausible ranges to explore the impact of these values on the results (Supplementary Table 1). The willingness to pay threshold of $50,000 per QALY gained was used to determine whether the intervention was cost-effective. This threshold is defined as the maximum amount society is willing to pay for an extra unit of health gain. Results from the univariate sensitivity analyses are presented as tornado plots (Fig. 2a–c). In this plot, each bar represents the impact of uncertainty in each variable on the incremental cost-effectiveness ratio.

**Figure 3:**
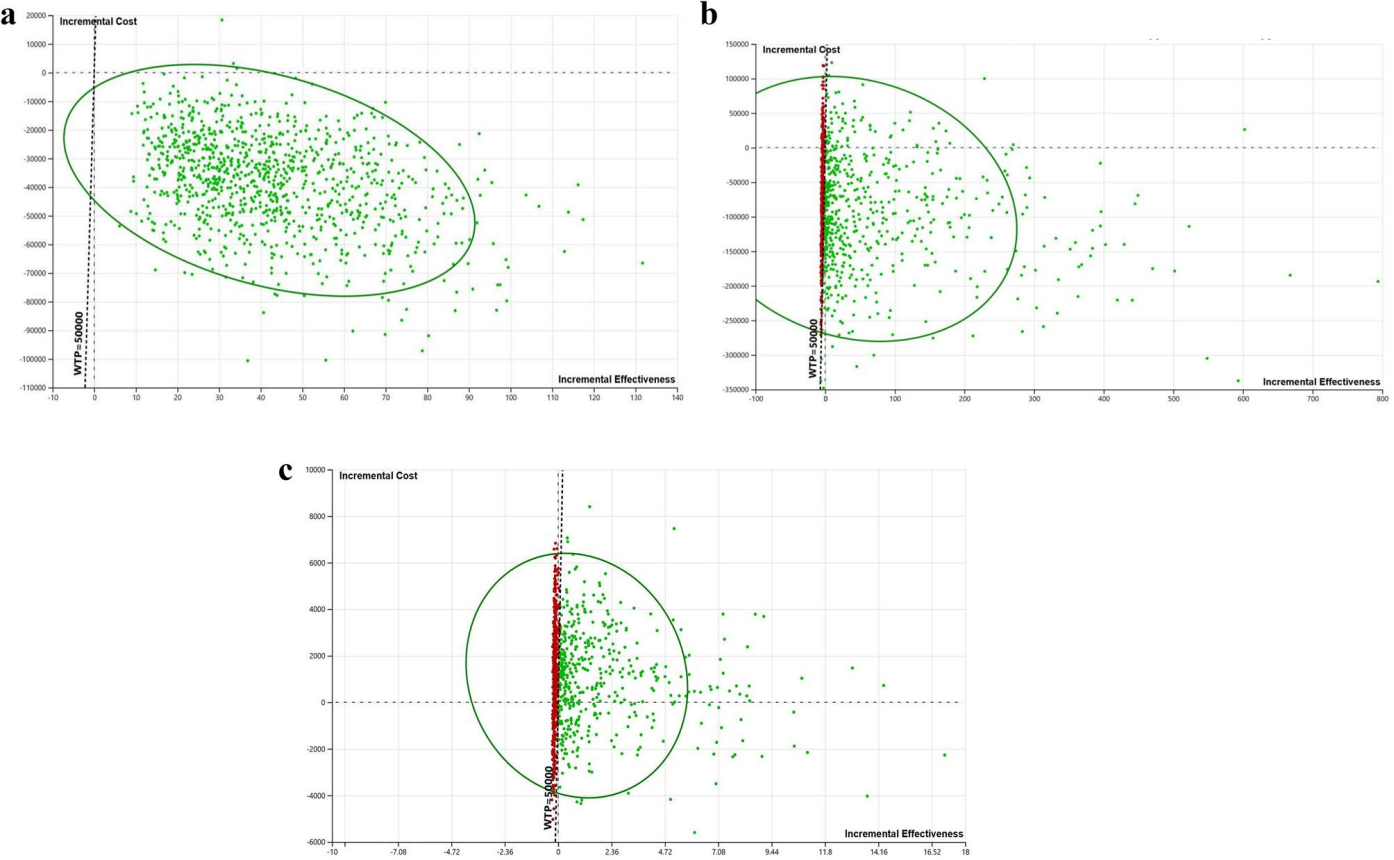
(**a**) Incremental cost-effectiveness scatterplot of 1000 samples comparing RGT to no RGT for *M. genitalium* infections in the cohort of 100,000 women with a willingness to pay threshold of $50,000/QALY gained. The green circle shows the 95% confidence interval. (**b**) Incremental cost-effectiveness scatterplot of 1000 samples comparing RGT to no RGT for *M. genitalium* infections in the cohort of 100,000 MSM with a willingness to pay threshold of $50,000/QALY gained. The green circle shows the 95% confidence interval. MSM, Men who have sex with men. (**c**) Incremental cost-effectiveness scatterplot of 1000 samples comparing RGT to no RGT for *M. genitalium* infections in the cohort of 100,000 MSW with a willingness to pay threshold of $50,000/QALY gained. The green circle shows the 95% confidence interval. MSW, Men who have sex with women.

In probabilistic sensitivity analysis (PSA), multiple key input parameters were varied simultaneously across the cohort of 100,000 women, MSM and MSW over 1000 simulations. Gamma distributions were applied for treatment costs, consultation visits, tests, and cumulative costs for *M. genitalium*-related complications. Beta distributions were applied for treatment cure rate, utility weight and proportion of cohorts tested and treated. The results of the PSA were presented as an incremental cost-effectiveness scatter plot (Fig. 3a–c) to visualise the distribution of PSA results of RGT and no RGT.

## Results

We evaluated the costs, population impact and cost-effectiveness of RGT compared to no RGT for treating *M. genitalium* infections in a modelled cohort of 100,000 women, 100,000 MSM and 100,000 MSW in Australia over ten years (Table 2).

**Table 2:** Model results for RGT and no RGT for treating *M. genitalium* infections in women, MSM and MSW. Total expected ten-year costs and effectiveness, simulation of a 100,000 people cohort (in $, 2022 prices, 3% annual discount rate).

Strategy	Costs ($ million, accumulated over ten years)				Population impact (accumulated over ten years)			Cost-effectiveness analysis				
	Total costs	Costs for diagnosis	Costs for treatment	Cost of complication	Effectiveness QALY (× 10^3^)	Number of symptomatic *M. genitalium* infections	Number of *M. genitalium-*related complications	Cost/ Effectiveness (C/E)	Incremental costs ($ × 10^3^)	Incremental QALY	Incremental NMB $ (at WTP threshold $50,000)	ICER ($/QALY gained)
Cohorts of women												
No RGT	0.75	0.17	0.05	0.53	996.76	1260	575	0.75	–	–		–
RGT	0.64	0.14	0.09	0.41	996.74	1250	435	0.65	− 104.25	28.93	1,342,250	− 3,604.01 (dominates, superior*)
Cohorts of MSM												
No RGT	2.22	1.67	0.55	Not conducted	1,026.61	13,828	Not conducted	2.17	–	–		–
RGT	2.07	1.33	0.73	Not conducted	1,026.97	13,252	Not conducted	2.01	− 156.63	361.78	17,932,372	− 432.94 (dominates, superior*)
Cohorts of MSW												
No RGT	0.45	0.34	0.10	Not conducted	991.26	2,977	Not conducted	0.45	–	–	–	–
RGT	0.45	0.29	0.16	Not conducted	991.23	2,931	Not conducted	0.46	6.4	− 32.92	− 1,652,484	− 196.96 (dominates inferior**)

ICER, Incremental Cost-Effectiveness Ratio; MSM, Men who have sex with men; MSW, Men who have sex with women; NMB, Net Monetary Benefit; QALY, Quality Adjusted Life Years; RGT, Resistance Guided Therapy; WTP, Willingness-To-Pay.

*The intervention alternative yields both lower costs and higher utility and is cost saving compared to no RGT.

**The intervention alternative yields both higher costs and lower utility compared to no RGT.

### Costs, effectiveness and cost-effectiveness of RGT in women

The total population being considered in this article is 300,000 (100,000 for each population group—women, MSM and MSW). Without RGT, the projected total costs of *M. genitalium* infections for 100,000 women over ten years were $0.75 million. The largest cost component was associated with complications from *M. genitalium* infections*.* The projected effectiveness for a cohort of 100,000 women over ten years was 996,736 QALYs. Without RGT, there were 575 *M. genitalium-*related cumulative complication events among 100,000 women in Australia over ten years.

Our model demonstrated that implementing RGT for treating *M. genitalium* infections in a cohort of 100,000 women in Australia over ten years was cost saving, resulting in lower costs and higher QALYs than no RGT. The total costs and effectiveness associated with RGT over ten years were $0.64 million and 996,765 QALYs, respectively. The net monetary benefit (NMB) of RGT at the WTP threshold of $50,000 is $1.3 million. The incremental cost-effectiveness ratio is -$3604 /QALY gained. The number of *M. genitalium-*related complication events over ten years was also lower by 140 compared to no RGT scenario.

### Costs, effectiveness and cost-effectiveness of RGT in MSM

Without RGT, the projected total costs of *M. genitalium* infections over ten years for a cohort of 100,000 MSM were $2.22 million, with the largest cost component being the costs for diagnosis. The projected effectiveness for a cohort of 100,000 MSM with no RGT was 1,026,662 QALYs gained.

Our model demonstrated that implementing RGT for treating *M. genitalium* infections in a cohort of 100,000 MSM in Australia over ten years was cost saving, resulting in lower costs and higher QALYs than no RGT. The total costs and effectiveness associated with RGT over ten years were $2.07 million and 1,026,974 QALYs, respectively. The NMB of RGT at the WTP threshold of $50,000 is $17.9 million. The incremental cost-effectiveness ratio was -$432.94/QALY gained.

### Costs, effectiveness and cost-effectiveness of RGT in MSW

Without RGT, the projected total costs of *M. genitalium* infections over ten years for a cohort of 100,000 MSW amounted to $0.45 million, with the largest cost component being the costs of diagnosis. The projected effectiveness for a cohort of 100,000 MSW with no RGT was 991,267 QALYs gained over ten years.

Our model demonstrated that implementing RGT for treating *M. genitalium* infections in a cohort of 100,000 MSW in Australia over ten years was not cost-effective at the WTP of $50,000, with an incremental cost-effectiveness ratio of -$196.96 per QALY gained. The total costs and effectiveness associated with RGT over ten years were $0.45 million and 991,234 QALYs, respectively, for a cohort of 100,000 MSW in Australia.

### Sensitivity analysis

Tornado plots were used to present the results of the univariate sensitivity analyses in Fig. 2a–c. The incremental cost-effectiveness ratio (ICER) for comparing RGT to no RGT for *M. genitalium* infections in women (Fig. 2a) was most sensitive to several factors: the utility during *M. genitalium* infections, the utility when experiencing *M. genitalium*-related complications, and the costs associated with *M. genitalium*-related complications.

The ICER for comparing RGT to no RGT for *M. genitalium* infections in MSM (Fig. 2b) was most sensitive to the cure rate of first-line therapy in no RGT, the cost of first-line therapy in RGT for those macrolides susceptible, and cost of second-line therapy in no RGT. . For MSW (Fig. 2c), the ICER for comparing RGT to no RGT for *M. genitalium* infections was most sensitive to the cure rate of first-line therapy in no RGT, the cost of first-line therapy in RGT for those macrolides susceptible, and the probability of getting treatment.

Figure 3a–c illustrate the results of the probability sensitivity analysis. Figure 3a demonstrates that RGT has a 100% probability of being cost-effective in women, while for MSM, RGT has an 88.2% probability of being cost-effective at the given willingness-to-pay threshold. Figure 3C shows that RGT has 60.7% probability of being cost-effective in MSW at the given WTP threshold (WTP = $50,000).

## Discussion

In this cost-effectiveness analysis, using RGT for treating *M. genitalium* infections in women and MSM proved to cost less, be more effective, and was associated with lower rates of complications compared to the no RGT. In MSW, while the use of RGT for determining the treatment of *M. genitalium* infections was not cost-effective, the PSA shows that there is a probability of 60.7% RGT could be cost-effective at the willingness-to-pay (WTP) threshold of $50,000. Our model also predicted that RGT would lead to fewer *M. genitalium* infections in women and MSM over ten years. This means that fewer infections are transmitted and fewer return visits and antibiotics are used, and indicates that RGT is likely to have additional, broader population benefits in slowing the future rise and spread of AMR in both target and non-target pathogens. For the MSW cohort, RGT did not result in fewer *M. genitalium* infections, which might be caused by the lower testing rate among this population group. Our findings support current Australian guidelines that RGT should be the recommended management strategy for *M. genitalium* infections and should prompt countries with similar parameters to adopt RGT to reduce healthcare costs and improve antibiotic stewardship.

Our univariate sensitivity analyses revealed the factors that could significantly impact the cost-effectiveness of RGT. Among women, two of the most influential factors for the ICER was associated with *M. genitalium*-related complications. Previous meta-analyses have demonstrated a twofold increased risk of cervicitis, PID, spontaneous abortion, and infertility in women infected with *M. genitalium*, which results in higher costs and a greater disease burden for affected women^10^. Our model only considered PID and chronic pelvic pain as *M. genitalium*-related complications in women due to data availability. While our model provides valuable insights, we acknowledge the need for more evidence around *M. genitalium*-related complications in women. Some complications remain areas of debate within the field, and further research is essential to address these uncertainties. Among MSM and MSW, the cure rate of the first-line therapy in no RGT has the most significant impact on the ICER. This highlights the likelihood of RGT being more cost-effective in these two populations with the increasing rate of macrolide resistance globally in the future.

Reducing antibiotic usage in treating *M. genitalium* is essential for addressing the global public health crisis of rising AMR among STIs^19^. *M. genitalium* has rapidly accumulated high rates of AMR, making it increasingly challenging to treat, especially against the backdrop of already scarce treatment options^28^. Our findings demonstrated that RGT was a cost-saving intervention that can contribute to slowing the rise of AMR by reducing antibiotic misuse and overuse. Studies from various settings have shown that RGT is easy to implement and tends to have high adherence, providing value for money^2,20^. However, widespread adoption of this strategy is hindered by the limited availability of diagnostic technology, associated costs, and treatment options for *M. genitalium* infections globally^29^.

Our study assumed a static antibiotic resistance profile over a ten-year time horizon, while a meta-analysis has shown a plateau in macrolide resistance in *M. genitalium* in Australia since 2017^30^. This interesting trend in macrolide resistance coincides with a reduction in using 1 g of azithromycin for *M. genitalium, C. trachomatis*, and STI syndromes in Australia, as well as the introduction of RGT for *M. genitalium.* Future studies on the cost-effectiveness analysis of RGT for *M. genitalium* infections may benefit from using a dynamic antibiotic resistance profile, expanding the analysis to a societal perspective, obtaining more robust estimates for variables that were identified as influential in our sensitivity analyses (e.g., for incidence of *M. genitalium*-related complications in women, probability of getting treatment), and assessing the epidemiological impact of antibiotic stewardship on the transmission dynamics of resistant *M. genitalium*. Next-generation RGT strategies that include fluoroquinolone resistance targets are also being developed, making it necessary to evaluate their cost-effectiveness across different sub-populations and geographical regions^28,31^.

Our study has several limitations that warrant consideration. First, the absence of publications on utility weights for individuals with *M. genitalium* infections and related complications prompted us to derive these values from studies on *C. trachomatis*, given the similarities in pathogenesis and clinical presentations of both infections^32^. We subjected these assumptions to sensitivity analyses to assess their potential impact on our conclusions. While they did not significantly affect our results in MSM and MSW, they were most influential in women, indicating that lower utility will have a significant impact on decreasing the ICER. Second, our study's findings may not be universally applicable, as the availability of molecular assays for resistance testing and alternative medications may vary across different regions. It would be beneficial to conduct economic evaluations tailored to specific settings and their unique management recommendations. Lastly, our model only calculated *M. genitalium*-related complications among cohorts of women, as limited evidence is available on the longer-term impact of *M. genitalium* infections among MSM and MSW. If complications such as epididymitis occurred, it would further favour using RGT in managing *M. genitalium* infections in MSM and MSW.

In conclusion, our study demonstrates that RGT is a cost-saving intervention, offering greater effectiveness and lower costs for women and MSM compared to no RGT. In MSW, RGT has probability of 60.7% to be cost-effective at the WTP threshold of $50,000. Future economic evaluations should be conducted in diverse settings with varying prevalence profiles and other subpopulations to validate and expand upon our findings.

### Supplementary Information

Supplementary Information.

## Data Availability

No datasets were generated during the current study. Data inputs were obtained from published studies or publicly available sources and are reported within this article and its supplementary material.
