# Supplementary material for: Cost-effectiveness of resistance-guided therapy for Mycoplasma genitalium in Australia
Source: Sci Rep. 2024 Jun 4;14:12856. doi: 10.1038/s41598-024-63056-1 (PMC11150248; doi:10.1038/s41598-024-63056-1)
Supplement: Supplementary file 1 — Supplementary Information. [file 41598_2024_63056_MOESM1_ESM.pdf]

Supplementary Materials

Supplement to: Cost-effectiveness of resistance-guided therapy for *Mycoplasma genitalium* in Australia

Table of contents

Supplementary Table 1. Input parameters and range for sensitivity analysis (events probabilities, cost and utility………2

Supplementary Table 2. Treatment strategy details for RGT and no RGT…………………………………………4

Supplementary Figure 1. Treeage model structure for RGT and no RGT arm……………………………………5

Supplementary Figure 2. Decision tree model for *M. genitalium*-related complications in women……9

Supplementary Table 3. Consolidated Health Economic Evaluation Reporting Standards (CHEERS) 2022 checklist………10

References………12

Supplementary table 1. Input parameters and range for sensitivity analysis (events probabilities, costs, and utility)

Parameters (unit)	Value	The range for sensitivity analyses	Ref	Included in univariate sensitivity analysis	Include d in PSA
The annual incidence rate of *M. genitalium* (per 100 person/year)	Women: 1·33 (0.355)<br>MSM: 6·63<br>Heterosexual men: 1·3	Women: 0·8 – 2·3<br>MSM: 6·63 – 29·5<br>MSW: 0·8 – 2·3	[1]<br>[2]	✓	X
Discount rate (%)	3 % (for all)	1 % - 5 %		✓	X
Time Horizon (years)	10 years = 520 cycles (1 cycle equal 1 week) (for all)	5 – 15 years<br>260 – 780 cycles (for all)		✓	X
Percentage of symptomatic *M. genitalium* infections (%)	Women: 6%<br>MSM: 8%<br>Heterosexual men: 5%	Not included	[3]<br>[4]<br>[5]	✓	X
Proportion cured using the first-line treatment (no RGT arm)	[Western Pacific]<br>Women: 0· 54<br>MSM: 0·31<br>[Globally]<br>Heterosexual men: 0·46	[Western Pacific]<br>Women: 95%CI (0·432 – 0·653)<br>MSM: 95%CI (0·15 - 0·48)<br>[Globally]<br>MSW: 95%CI (0·33 – 0·60)	[6]	✓	✓
Proportion cured using the second-line treatment (no RGT arm)	0·85 (for all)	95% CI (0·8 – 0·89) (for all)	[7]	✓	✓
Proportion cured using the first-line treatment (RGT arm)	0·942 (for all)	95% CI (0·87 – 0·98) (for all)	[9]	✓	✓
Proportion cured using the second line treatment (RGT arm)	0·71 (for all)	95% CI (0·54 – 0·85) (for all)	[8]	✓	✓
Proportion of symptomatic *M. genitalium* infections that are getting tested	Women: 0·54<br>MSM: 0·66<br>Heterosexual men: 0.47	Women: ±30%, 0·24 – 0·84<br>MSM: ±30%, 0·46 – 0·86<br>MSW: ±30%, 0·33 – 0·61	[10]	✓	✓
Proportion of cohorts that are getting treated if diagnosed	Women: 0·80<br>MSM: 0·77<br>Heterosexual men: 0.80	Women: 0·8 – 1·0<br>MSM: 0·72 – 0·82<br>MSW: 0·8 – 1·0	[11]<br>[12]<br>[11]	✓	✓
Probability of women cohorts that got *M. genitalium*-related complications	0·025	±30% (0·0175 – 0·0325)	Calculation	✓	✓
Spontaneous clearance rate (per week)	Women: 0·015<br>MSM: 0·02<br>MSW: 0.02	Women: 0·01-0·2<br>MSM: 0·01-0·2<br>MSW: 0·01-0·2	[13]	X	X
**Costs input**	**Value** (in 2022 prices, Australian Dollar AUD)	**Range for sensitivity analyses**	**Ref**		
Cost of GP visit	AU$ 38·75 (for all)	±30% (AU$ 27·125 – 50·375)	MBS online	✓	✓
Cost to diagnose *M. genitalium* infections	AU$ 28·65 (for all)	±30% (AU$ 20·1 – 37·25)	MBS online	✓	✓
Cost of resistance test	AU$ 11 (for all)	±30% (AU$ 7·7 – 14·3)	MBS online	✓	✓
Cost of 1st line treatment in no RGT	AU$ 17·38 (for all)	±30% (AU$ 12·2 – 22·6)	PBS online	✓	✓
Cost of 2nd line treatment in no RGT	AU$ 86·10 (for all)	±30% (AU$ 60·27 – 111·93)	PBS online [15]	✓	✓

Cost of 1st line in RGT	Macrolide resistance:<br>AU$ 107·15 (for all)<br>Macrolide susceptible:<br>AU$ 73·19 (for all)	±30% (AU$ 75·01 – 139·64)<br><br>±30% (AU$ 51·23- 95·15)	PBS online [15]	✓	✓
Cost of 2nd line in RGT<br>A proportion of those received Moxifloxacin and Minocycline	Calculated<br>0·37(cost of Moxifloxacin)<br>+0·63(cost of Minocycline)<br>AU$ 45·80 (for all)	±30% (AU$ 32·06 - 59·54)	PBS online Calculation	✓	✓
Cumulative costs of *M. genitalium*-related complications	AU$ 1,083·4 (for all)	±30% (AU$ 758·4 – 1408·5)	Calculation	✓	✓
**Utility input**	**Value**	**Range for sensitivity analyses**	**References**		
Utility during *M. genitalium* infections	Women: 0·96<br>MSM: 0·96<br>Heterosexual men: 0·96	Women: ±30% (0·67 – 1·0)<br>MSM: ±30% (0·67 – 1·0)<br>MSW: ±30% (0·67 – 1·0)	[4]	✓	✓
Utility of *M. genitalium*-related complications	0·63	±30% (0·44 – 0·82)	Calculation	✓	✓

Notes:
CI: Confidence Interval; MBS: Medicare Benefit Scheme; MSM: Men who have sex with men; MSW: Men who have sex with women; PBS: Pharmaceutical Benefit Scheme PSA: Probability Sensitivity Analysis; RGT: Resistance-Guided Therapy

Supplementary table 2. Treatment strategy details for RGT and no RGT

**Intervention**	**No RGT**	**RGT**
First-line therapy	1 gram of azithromycin on day 1	**Macrolide susceptible**<br><br>Doxycycline 100 milligram twice a day for 7 days followed by 4 days of azithromycin; 1 gram in day 1 and 500 milligram in day 2 to 4 (total 2.5 gram)<br><br>**Macrolide Resistance**<br><br>7 days of doxycycline 100 milligram twice a day followed by 7 days of moxifloxacin 400 milligram daily for 7 days
Second-line therapy	7 days of moxifloxacin 400 milligram once a day	**For those who failed 1st line strategy and macrolide susceptible:**<br>7 days of moxifloxacin 400 milligram once a day<br><br>**For those who failed 1st line strategy and macrolide resistance:**<br>Minocycline 100 milligram twice a day for 14 days

Notes:
RGT: Resistance-Guided Therapy

Supplement figure 1. Treeage model structure for no RGT and RGT (Example from women model, with slight changes on MSM and MSW since they have no MG-related complications)

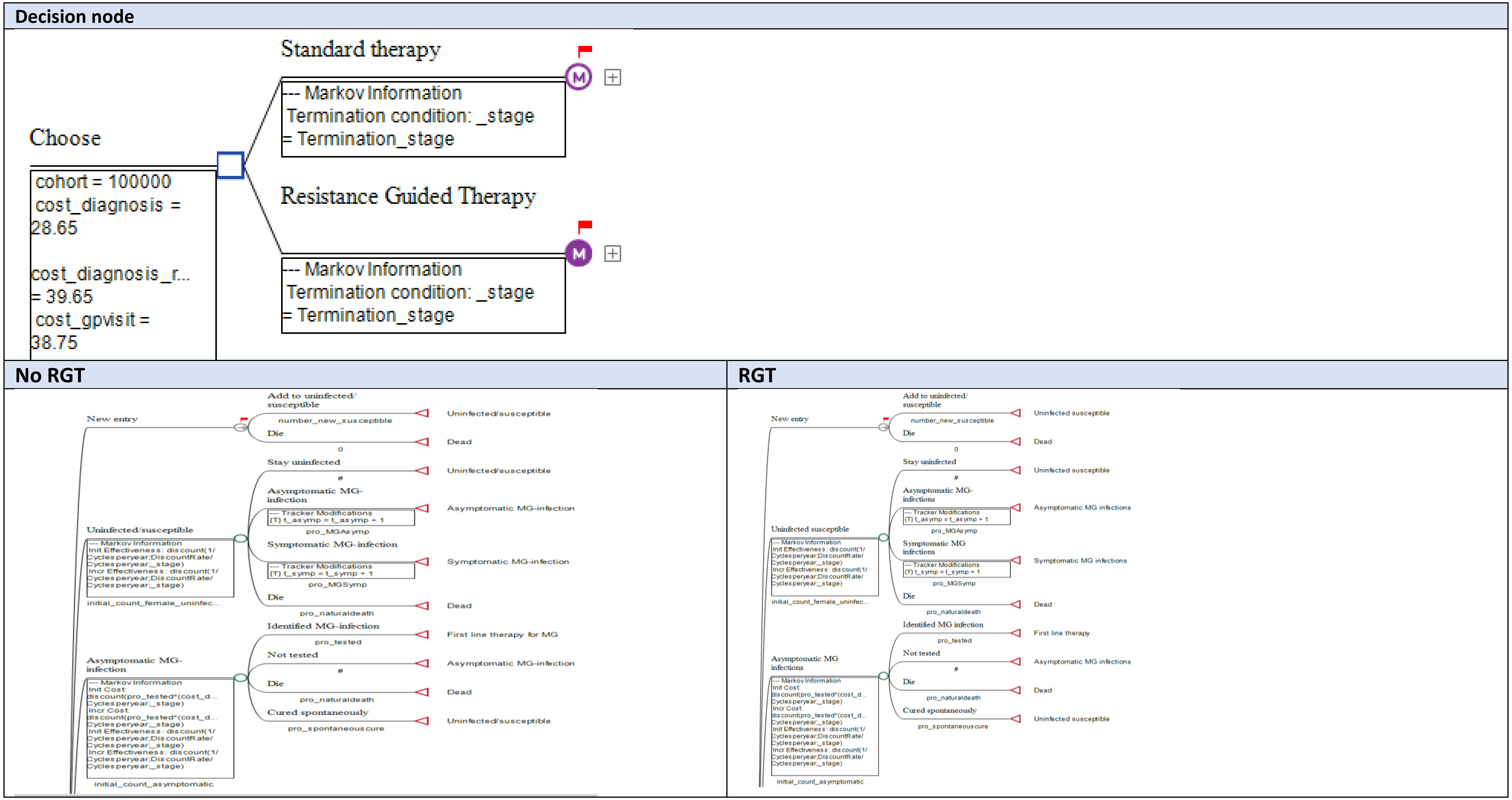

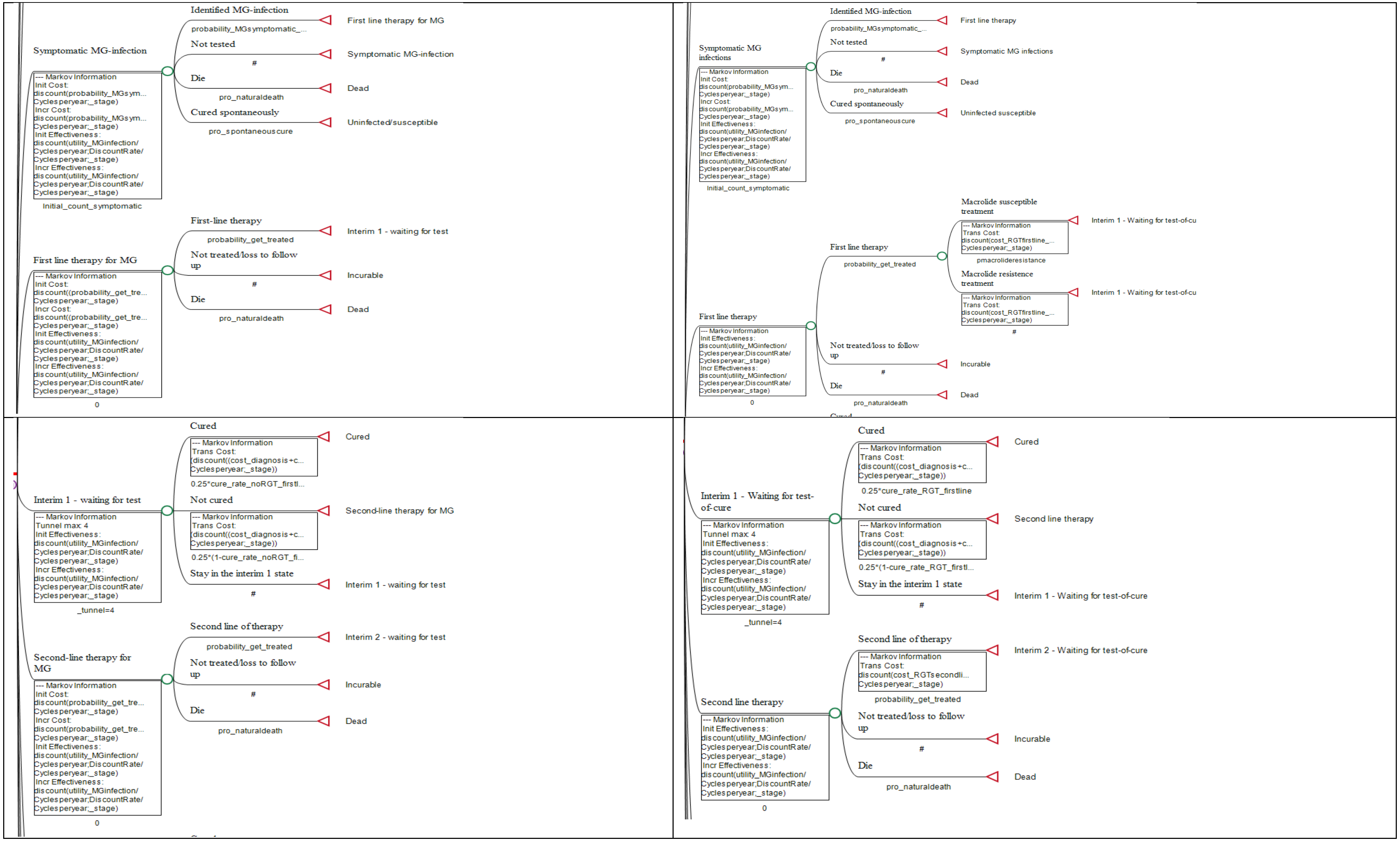
Symptomatic MG-infection
--- Markov Information
Init Cost:
discount(probability_MGsym...
Cyclesperyear;_stage)
Incr Cost:
discount(probability_MGsym...
Cyclesperyear;_stage)
Init Effectiveness:
discount(utility_MGinfection/
Cyclesperyear;DiscountRate/
Cyclesperyear;_stage)
Incr Effectiveness:
discount(utility_MGinfection/
Cyclesperyear;DiscountRate/
Cyclesperyear;_stage)
Initial_count_symptomatic
Identified MG-infection
probability_MGsymptomatic_...
First line therapy for MG
Not tested
#
Symptomatic MG-infection
Die
pro_naturaldeath
Dead
Cured spontaneously
pro_spontaneouscure
Uninfected/susceptible
First line therapy for MG
--- Markov Information
Init Cost:
discount((probability_get_tre...
Cyclesperyear;_stage)
Incr Cost:
discount((probability_get_tre...
Cyclesperyear;_stage)
Init Effectiveness:
discount(utility_MGinfection/
Cyclesperyear;DiscountRate/
Cyclesperyear;_stage)
Incr Effectiveness:
discount(utility_MGinfection/
Cyclesperyear;DiscountRate/
Cyclesperyear;_stage)
0
First-line therapy
probability_get_treated
Interim 1 - waiting for test
Not treated/loss to follow up
#
Incurable
Die
pro_naturaldeath
Dead
Symptomatic MG infections
--- Markov Information
Init Cost:
discount(probability_MGsym...
Cyclesperyear;_stage)
Incr Cost:
discount(probability_MGsym...
Cyclesperyear;_stage)
Init Effectiveness:
discount(utility_MGinfection/
Cyclesperyear;DiscountRate/
Cyclesperyear;_stage)
Incr Effectiveness:
discount(utility_MGinfection/
Cyclesperyear;DiscountRate/
Cyclesperyear;_stage)
Initial_count_symptomatic
Identified MG-infection
probability_MGsymptomatic_...
First line therapy
Not tested
#
Symptomatic MG infections
Die
pro_naturaldeath
Dead
Cured spontaneously
pro_spontaneouscure
Uninfected susceptible
First line therapy
--- Markov Information
Init Effectiveness:
discount(utility_MGinfection/
Cyclesperyear;DiscountRate/
Cyclesperyear;_stage)
Incr Effectiveness:
discount(utility_MGinfection/
Cyclesperyear;DiscountRate/
Cyclesperyear;_stage)
0
First line therapy
probability_get_treated
Macrolide susceptible treatment
--- Markov Information
Trans Cost:
discount(cost_RGTfirstline_...
Cyclesperyear;_stage)
pmacrolideresistance
Interim 1 - Waiting for test-of-cu
Macrolide resistence treatment
--- Markov Information
Trans Cost:
discount(cost_RGTfirstline_...
Cyclesperyear;_stage)
#
Interim 1 - Waiting for test-of-cu
Not treated/loss to follow up
#
Incurable
Die
pro_naturaldeath
Dead
Interim 1 - waiting for test
--- Markov Information
Tunnel max: 4
Init Effectiveness:
discount(utility_MGinfection/
Cyclesperyear;DiscountRate/
Cyclesperyear;_stage)
Incr Effectiveness:
discount(utility_MGinfection/
Cyclesperyear;DiscountRate/
Cyclesperyear;_stage)
_tunnel=4
Cured
--- Markov Information
Trans Cost:
(discount((cost_diagnosis+c...
Cyclesperyear;_stage))
0.25*cure_rate_noRGT_firstl...
Cured
Not cured
--- Markov Information
Trans Cost:
(discount((cost_diagnosis+c...
Cyclesperyear;_stage))
0.25*(1-cure_rate_noRGT_fi...
Second-line therapy for MG
Stay in the interim 1 state
#
Interim 1 - waiting for test
Second-line therapy for MG
--- Markov Information
Init Cost:
discount(probability_get_tre...
Cyclesperyear;_stage)
Incr Cost:
discount(probability_get_tre...
Cyclesperyear;_stage)
Init Effectiveness:
discount(utility_MGinfection/
Cyclesperyear;DiscountRate/
Cyclesperyear;_stage)
Incr Effectiveness:
discount(utility_MGinfection/
Cyclesperyear;DiscountRate/
Cyclesperyear;_stage)
0
Second line of therapy
probability_get_treated
Interim 2 - waiting for test
Not treated/loss to follow up
#
Incurable
Die
pro_naturaldeath
Dead
Interim 1 - Waiting for test-of-cure
--- Markov Information
Tunnel max: 4
Init Effectiveness:
discount(utility_MGinfection/
Cyclesperyear;DiscountRate/
Cyclesperyear;_stage)
Incr Effectiveness:
discount(utility_MGinfection/
Cyclesperyear;DiscountRate/
Cyclesperyear;_stage)
_tunnel=4
Cured
--- Markov Information
Trans Cost:
(discount((cost_diagnosis+c...
Cyclesperyear;_stage))
0.25*cure_rate_RGT_firstline
Cured
Not cured
--- Markov Information
Trans Cost:
(discount((cost_diagnosis+c...
Cyclesperyear;_stage))
0.25*(1-cure_rate_RGT_firstl...
Second line therapy
Stay in the interim 1 state
#
Interim 1 - Waiting for test-of-cure
Second line therapy
--- Markov Information
Init Effectiveness:
discount(utility_MGinfection/
Cyclesperyear;DiscountRate/
Cyclesperyear;_stage)
Incr Effectiveness:
discount(utility_MGinfection/
Cyclesperyear;DiscountRate/
Cyclesperyear;_stage)
0
Second line of therapy
--- Markov Information
Trans Cost:
discount(cost_RGTsecondli...
Cyclesperyear;_stage)
probability_get_treated
Interim 2 - Waiting for test-of-cure
Not treated/loss to follow up
#
Incurable
Die
pro_naturaldeath
Dead

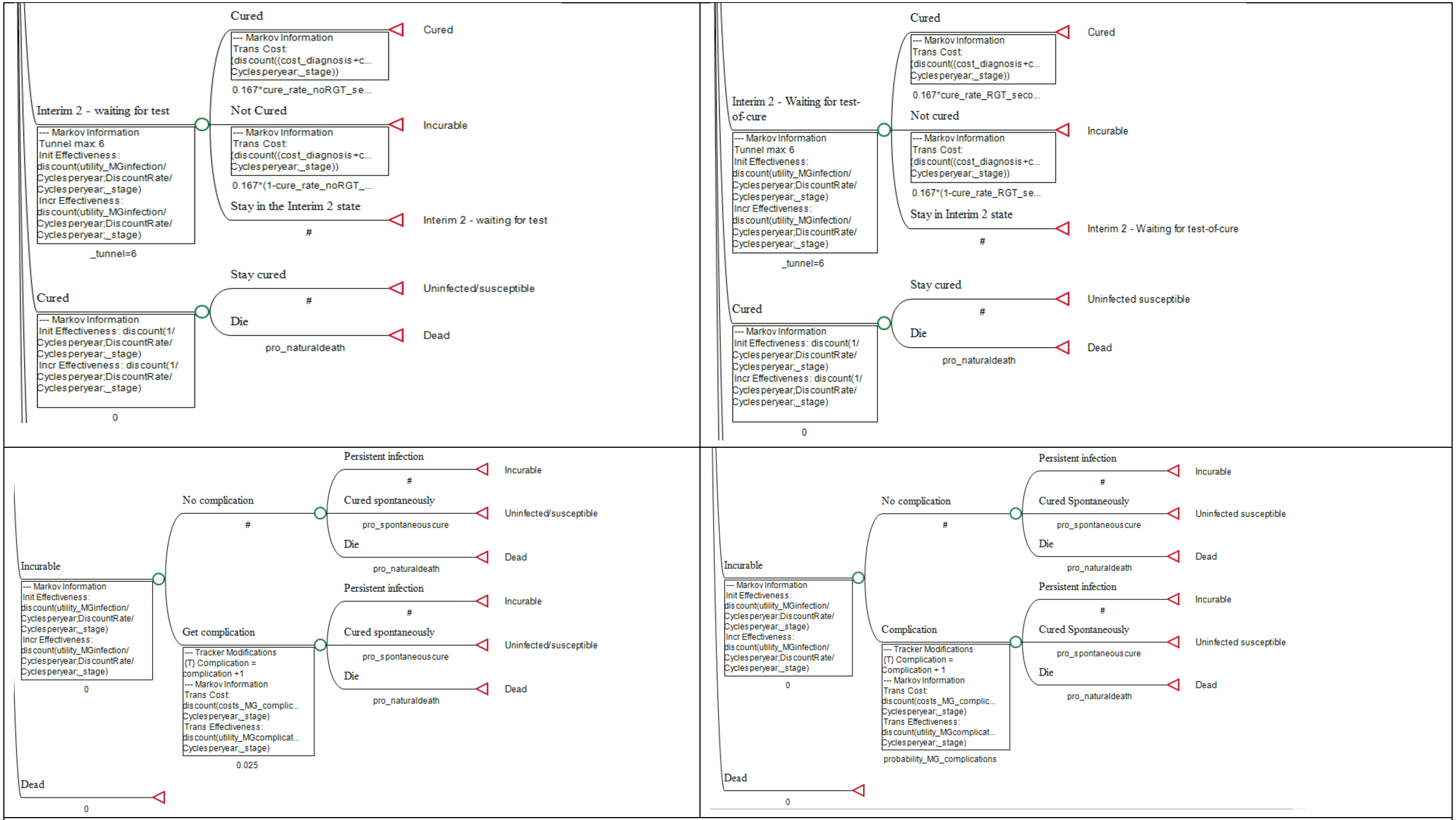

Notes:

AMR: Antimicrobial resistance; c_complications: Costs of *M. genitalium*-related complications in women; c_diagnosis: cost for diagnosis of *M. genitalium* infection; c_gpvisit: cost of one-time general practitioner (GP) visit; c_int1stline: cost of first-line therapy in RGT intervention; c_int2ndline: cost of second-line therapy in RGT intervention; c_int3rdline: cost of third-line therapy in RGT

intervention; c_std1stline: cost of first-line therapy in standard-of-care; c_std2ndline: cost of second-line therapy in standard-of-care; c_std3rdline: cost of third-line therapy in standard-of-care; c_resistance: cost for macrolide resistance test; CPP: Chronic pelvic pain; DiscountRate: Discount rate; EV: Expected value; ICE: Incremental Cost-Effectiveness; ICER: Incremental Cost-Effectiveness Ratio; MSM: Men who have sex with men; MBS: Medicare Benefits Schedule; NGU: non-*gonococcal* urethritis; PBS: Pharmaceutical Benefits Schedule; pcuredstd1stline: cure rate for the first-line therapy of standard-of-care; pcuredstd2ndline: cure rate for the second-line therapy of standard-of-care; pcuredstd3rdline: cure rate for the third-line therapy of standard-of-care; pro_complication: probability of getting *M. genitalium*-related complications; pro_tested_symp: probability of getting tested among people with symptomatic *M. genitalium* infection; pro_treatment: probability of people *with M. genitalium* infection to get treated; pro_cured_1stRGT: cure rate for the first-line therapy in RGT intervention; pro_cured_2ndRGT: cure rate for the second-line therapy in RGT intervention; pro_cured_3rdRGT: cure rate for the third-line therapy in RGT intervention; PSA: Probability sensitivity analysis; PID: Pelvic inflammatory disease; QALY: Quality adjusted life years; rate_MG: force of infection; RGT: Resistance-guided therapy; termination_stage: number of total cycles; u_complication: Utility of people with *M. genitalium*-related complications; u_mg: Utility of people with symptomatic *M. genitalium* infections; WTP: Willingness-to-pay

Supplementary figure 2. Decision tree model for *M. genitalium*-related complications in women

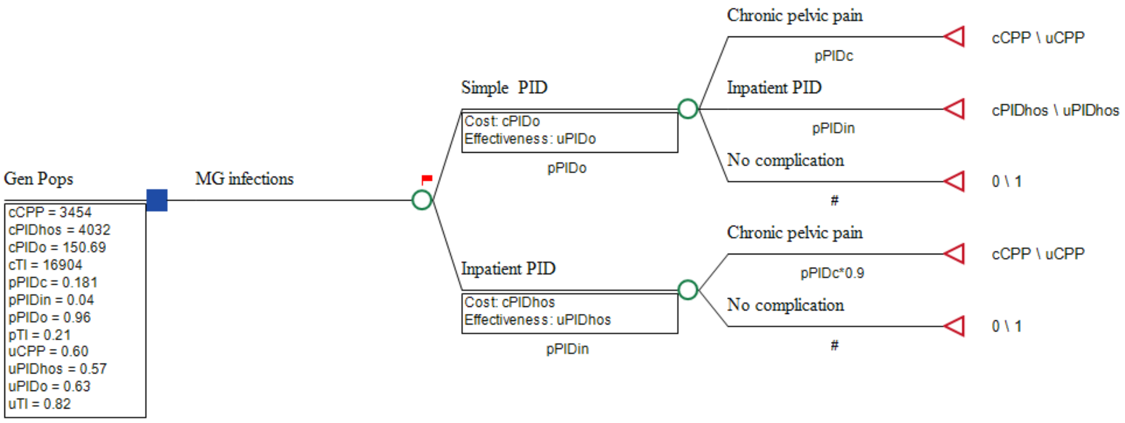

Notes:
cCPP: Average costs of chronic pelvic pain; cPIDhos: Average cost of inpatient pelvic inflammatory disease; cPIDo: Average cost of outpatient pelvic inflammatory disease; cTI: Average cost of tubal infertility; pPIDc: probability of getting chronic pelvic pain after PID-event; pPIDin: probability of getting inpatient pelvic inflammatory disease; pPIDo: probability of getting outpatient pelvic inflammatory disease; pTI; probability of getting tubal infertility; uCPP: utility of woman with chronic pelvic pain; uPIDhos: utility of woman with inpatient pelvic inflammatory disease; uPIDo: utility of woman with outpatient pelvic inflammatory disease; uTI: utility of woman with tubal infertility

Supplementary table 3. Consolidated Health Economic Evaluation Reporting Standards (CHEERS) 2022 checklist[14]

Topic	No	Item	Location where item is reported
Title	1	Identify the study as an economic evaluation and specify the interventions being compared.	1
Abstract		Provide a structured summary that highlights context, key methods, results, and alternative analyses.	2
Introduction			
Background and objectives	3	Give the context for the study, the study question, and its practical relevance for decision making in policy or practice	3-4
Methods			
Study population	4	Describe characteristics of the study population (such as age range, demographics, socioeconomic, or clinical characteristics).	4
Setting and location	5	Provide relevant contextual information that may influence findings.	4
Comparators	6	Describe the interventions or strategies being compared and why chosen.	5
Perspective	7	State the perspective(s) adopted by the study and why chosen	5
Time horizon	8	State the time horizon for the study and why appropriate	4-5
Discount rate	9	Report the discount rate(s) and reason chosen	5
Selection of outcome	10	Describe what outcomes were used as the measure(s) of benefit(s) and harm(s).	5
Measurement of outcome	11	Describe how outcomes used to capture benefit(s) and harm(s) were measured.	5
Valuation of outcomes	12	Describe the population and methods used to measure and value outcomes.	5
Measurement and valuation of resources and costs	13	Describe how costs were valued	5
Currency, price date, and conversion	14	Report the dates of the estimated resource quantities and unit costs, plus the currency and year of conversion	5
Rationale and description of model	15	If modelling is used, describe in detail and why used. Report if the model is publicly available and where it can be accessed	4
Analytics and assumptions	16	Describe any methods for analysing or statistically transforming data, any extrapolation methods, and approaches for validating any model used.	4-5
Characterising heterogeneity	17	Describe any methods used for estimating how the results of the study vary for subgroups.	5
Characterising distributional effects	18	Describe how impacts are distributed across different individuals or adjustments made to reflect priority populations	5
Characterising uncertainty	19	Describe methods to characterise any sources of uncertainty in the analysis.	5
Approach to engagement with patients and others affected by the study	20	Describe any approaches to engage patients or service recipients, the general public, communities, or stakeholders (such as clinicians or payers) in the design of the study	NA
Results			

Study parameters	21	Report all analytic inputs (such as values, ranges, references) including uncertainty or distributional assumptions.	Appendix p 2-3
Summary of main results	22	Report the mean values for the main categories of costs and outcomes of interest and summarise them in the most appropriate overall measure.	7
Effect of uncertainty	23	Describe how uncertainty about analytic judgments, inputs, or projections affect findings. Report the effect of choice of discount rate and time horizon, if applicable.	9-10
Effect of engagement with patients and others affected by the study	24	Report on any difference patient/service recipient, general public, community, or stakeholder involvement made to the approach or findings of the study	NA
Discussion			
Study findings, limitations, generalisability, and current knowledge	25	Report key findings, limitations, ethical or equity considerations not captured, and how these could affect patients, policy, or practice.	12-13
Other relevant information			
Source of funding	26	Describe how the study was funded and any role of the funder in the identification, design, conduct, and reporting of the analysis	2
Conflicts of interest	27	Report authors conflicts of interest according to journal or International Committee of Medical Journal Editors requirements.	14

References:

1 Walker J, Fairley CK, Bradshaw CS, Tabrizi SN, Twin J, Chen MY *et al.* Mycoplasma genitalium incidence, organism load, and treatment failure in a cohort of young Australian women. *Clin Infect Dis* 2013; **56**: 1094–1100.

2 Xu X, Bradshaw CS, Chow EPF, Ong JJ, Hocking JS, Fairley CK *et al.* Modelling the multiple anatomical site transmission of Mycoplasma genitalium among men who have sex with men in Australia. *Sci Reports 2021 111* 2021; **11**: 1–8.

3 Facts about chlamydia. https://www.ecdc.europa.eu/en/chlamydia/facts (accessed 20 Nov2022).

4 Read TRH, Fairley CK, Murray GL, Jensen JS, Danielewski J, Worthington K *et al.* Impact of screening on the prevalence and incidence of Mycoplasma genitalium and its macrolide resistance in men who have sex with men living in Australia: A mathematical model. *EClinicalMedicine* 2021; **33**. doi:10.1016/J.ECLINM.2021.100779/ATTACHMENT/1F5B9325-A64E-4904-B53A-4136AB5C1992/MMC1.DOCX.

5 Horner PJ, Martin DH. Mycoplasma genitalium Infection in Men. *J Infect Dis* 2017; **216**: S396.

6 Machalek DA, Tao Y, Shilling H, Jensen JS, Unemo M, Murray G *et al.* Prevalence of mutations associated with resistance to macrolides and fluoroquinolones in Mycoplasma genitalium: a systematic review and meta-analysis. *Lancet Infect Dis* 2020; **20**: 1302–1314.

7 Vodstrcil LA, Plummer EL, Doyle M, Murray GL, Bodiyabadu K, Jensen JS *et al.* Combination Therapy for Mycoplasma genitalium, and New Insights Into the Utility of parC Mutant Detection to Improve Cure. *Clin Infect Dis* 2022; **75**: 813–823.

8 Doyle M, Vodstrcil LA, Plummer EL, Aguirre I, Fairley CK, Bradshaw CS. Nonquinolone options for the treatment of Mycoplasma genitalium in the era of increased resistance. *Open Forum Infect Dis* 2020; **7**. doi:10.1093/ofid/ofaa291.

9 Read TRH, Fairley CK, Murray GL, Jensen JS, Danielewski J, Worthington K *et al.* Clinical Infectious Diseases Outcomes of Resistance-guided Sequential Treatment of Mycoplasma genitalium Infections: A Prospective Evaluation. *Clin Infect Dis* 2019; **68**: 554–560.

10 Farquharson R, Fairley C, Abraham E, Bradshaw C, Plummer E, Ong J *et al.* Time to healthcare-seeking following the onset of symptoms among men and women attending a sexual health clinic in Melbourne, Australia. *Front Med* 2022; **0**: A5.2-A6.

11 Owusu-Edusei K, Chesson HW, Gift TL, Brunham RC, Bolan G. Cost-effectiveness of Chlamydia Vaccination Programs for Young Women. *Emerg Infect Dis* 2015; **21**: 960.

12 Latimer RL, Shilling HS, Vodstrcil LA, Machalek DA, Fairley CK, Chow EPF *et al.* Prevalence of Mycoplasma genitalium by anatomical site in men who have sex with men: a systematic review and meta-analysis. *Sex Transm Infect* 2020; **96**: 563–570.

13 Ong JJ, Ruan L, Lim AG, Bradshaw CS, Taylor-Robinson D, Unemo M *et al.* Impact of screening on the prevalence and incidence of Mycoplasma genitalium and its macrolide resistance in men who have sex with men living in Australia: A mathematical model. *EClinicalMedicine* 2021; **33**. doi:10.1016/j.eclinm.2021.100779.

14 Husereau D, Drummond MF, Augustovski F, Bekker-Grob E De, Briggs AH, Carswell C *et al.* Consolidated Health Economic Evaluation Reporting Standards 2022 (CHEERS 2022) statement: updated reporting guidance for health economic evaluations. *BM Med* 2023; **20**.

15. Ong JJ, Lim A, Bradshaw C, Taylor-Robinson D, Unemo M, Horner PJ, Vickerman P, Zhang L. Cost-effectiveness of testing for Mycoplasma genitalium among men who have sex with men in Australia. *Sex Transm Infect*. 2023 Aug 17;**99**(6):398-403. doi: 10.1136/sextrans-2022-055611. PMID: 36958826.
